# Simple method for high-performance stretchable composite conductors with entrapped air bubbles

**DOI:** 10.1186/s11671-016-1229-8

**Published:** 2016-01-12

**Authors:** Hyejin Hwang, Dae-Gon Kim, Nam-Su Jang, Jeong-Ho Kong, Jong-Man Kim

**Affiliations:** Department of Nanomechatronics Engineering, Pusan National University, Busan, 609-735 Republic of Korea; Department of Nano Fusion Technology and BK21 Plus Nano Convergence Technology Division, Pusan National University, Busan, 609-735 Republic of Korea; Department of Nanoenergy Engineering, Pusan National University, Busan, 609-735 Republic of Korea; Present address: Department of Nanoscience and Technology, Seoul National University, Seoul, 151-742 Republic of Korea; Present address: School of Electrical and Computer Engineering, Seoul National University, Seoul, 151-742 Republic of Korea

**Keywords:** Entrapped air bubbles, Conductive elastic composites, Surfactant, Stretchable conductor, CNT networks

## Abstract

We integrate air bubbles into conductive elastic composite-based stretchable conductors to make them mechanically less stiff and electrically more robust against physical deformations. A surfactant facilitates both the formation and maintenance of air bubbles inside the elastic composites, leading to a simple fabrication of bubble-entrapped stretchable conductors. Based on the unique bubble-entrapped architecture, the elastic properties are greatly enhanced and the resistance change in response to tensile strains can clearly be controlled. The bubble-entrapped conductor achieves ~80 % elongation at ~3.4 times lower stress and ~44.8 % smaller change in the electrical resistance at 80 % tensile strain, compared to bare conductor without air bubbles.

## Background

Conductive elastic materials that can retain their electrical performance under various deformations have recently received great attention due to the potential applications as stretchable conductors and interconnects in stretchable electronics [1–5]. Many efforts have been made to demonstrate various types of conductive elastic materials. Examples include electrical nanonetworks mainly made of conductive carbon nanotubes (CNTs) [6–8] and metallic nanowires (NWs) [9–11], buckled architectures of metallic thin films [12–14], three-dimensional conductive foams [15–17], and conductive composites synthesized by doping elastomer matrices with conductive nanofillers [18–22].

Among these methods, conductive composites have been considered as one of the most efficient ways of preparing conductive elastic materials due to the superior advantages including fabrication simplicity and easy control of the intrinsic electrical properties. In this regard, to date, this method has been widely used to fabricate functional stretchable devices. However, two critical issues can potentially arise in the conductive composite-based approaches: (1) considerable increase of the stiffness of the composites from excessive filler doping and (2) steep change in the electrical conductivity when stretched. These would lead to significant degradation of the mechanical and electrical performance of the stretchable devices.

In this work, we present a new class of CNT-doped stretchable conductor with embedded air bubbles (hereafter, bubble conductor) based on conductive elastic composites. The air bubbles inside the composite matrix play a key role in (1) decreasing the stiffness of the composites and (2) sustaining the electrical properties, mainly by allowing considerable reduction of the resistance change of the CNT network located at the boundary regions upon stretching. The advantages of the proposed approach include process simplicity, cost-effectiveness, and potential scalability for large area fabrication.

## Methods

### Fabrication of Bubble Conductors

**Fig. 1:**
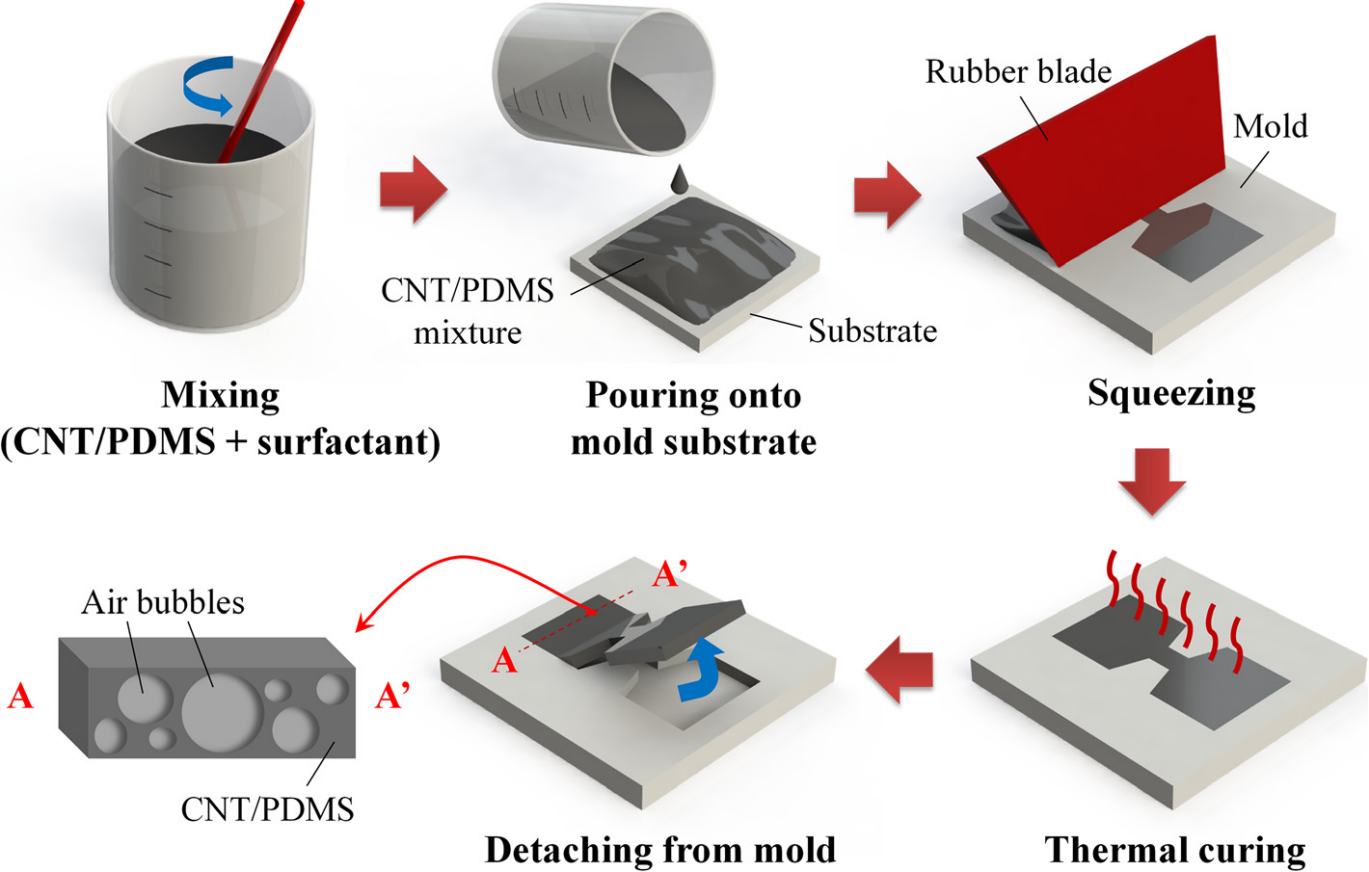
Schematic illustration of fabrication sequence for bubble-entrapped stretchable conductor

The fabrication process of the proposed bubble conductors is schematically illustrated in Fig. 1. Conductive elastic composites with air bubbles were first synthesized by dispersing ~3 wt% multiwalled CNTs in polydimethylsiloxane (PDMS; Sylgard 184, Dow Corning) prepolymer mixed with a curing agent at a weight ratio of 10:1, followed by manually stirring for 30 min. A small quantity of surfactant (sodium lauroyl oat amino acid) was added to the CNT/PDMS mixture to facilitate high-density bubble formation and retainment in the mixture. The surfactant-added CNT/PDMS mixture was then squeezed selectively onto a silicon substrate using a polymeric mold with a height of ~500 μm. Finally, the bubble conductors were peeled off from the substrate after thermally curing on a hotplate at 80 °C for 30 min. The bare conductors without air bubbles were fabricated by the abovementioned procedure except adding the surfactant.

### Characterization

The surface morphologies of the fabricated bubble conductors were observed using an optical microscope (OM; BX60M, OLYMPUS) equipped with a CCD module. A field emission scanning electron microscope (FESEM; S4700, HITACHI) was used to characterize the detailed morphologies of the air bubbles and CNT networks in the conductors. The stress-strain relationships of the bare conductor and bubble conductor were examined using an automatic testing stand (JSV-H1000, JISC) equipped with a digital push-pull force gauge (HF-10, JISC). All stretching tests were performed using a custom-made jig while monitoring the change in the electrical resistance using a digital multimeter (U1253B, Agilent Technologies). The electrical resistances of the conductors under stretching were recorded after imposing a stabilization time of 20 s in each strained state.

## Results and Discussion

**Fig. 2:**
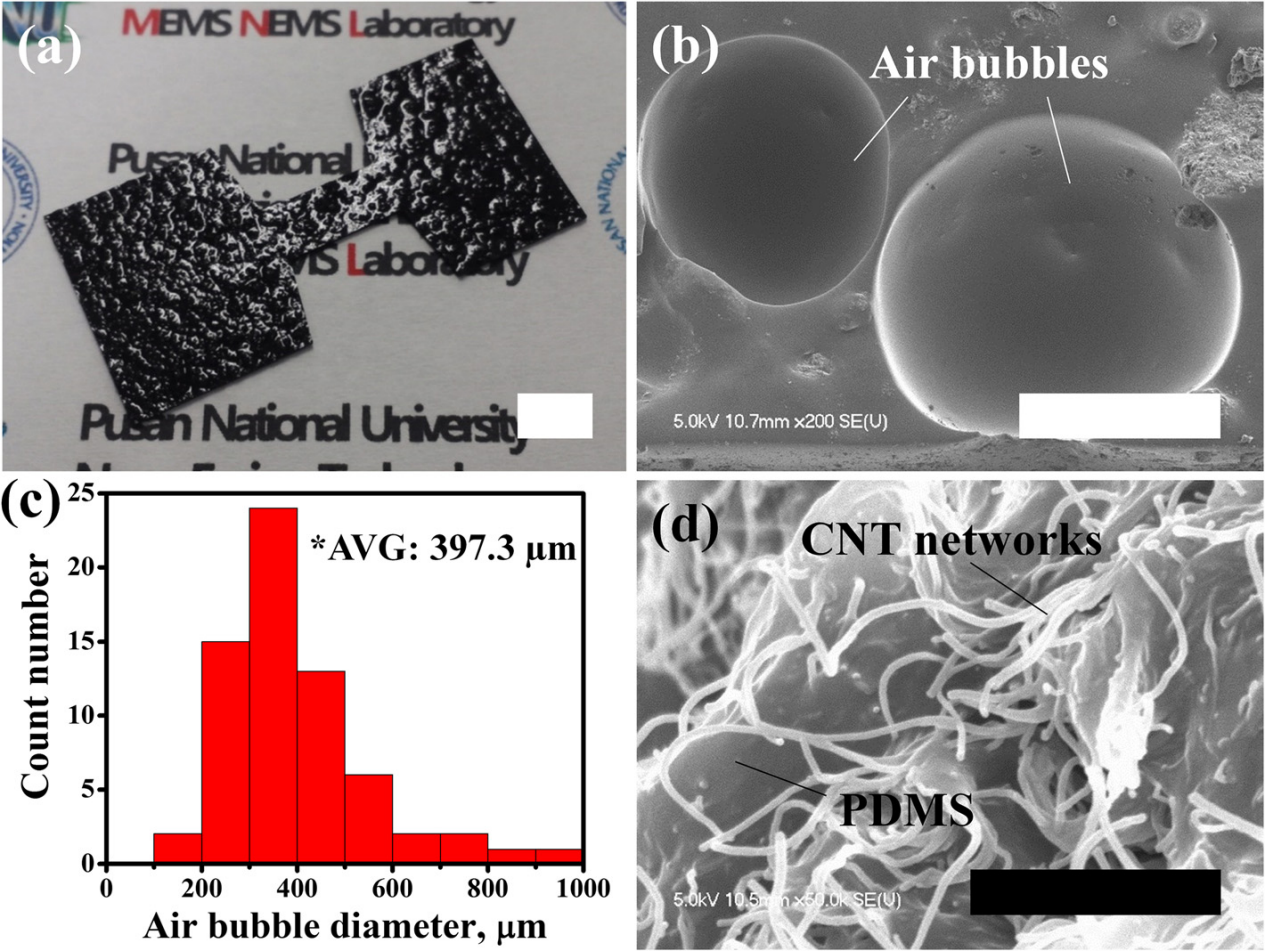
Fabrication results. **a** Digital image of the fabricated bubble conductor (*scale bar*: 1 cm), **b** cross-sectional SEM image (*scale bar*: 200 μm) and **c** diameter distribution (66 air bubbles measured) of air bubbles in the CNT-doped PDMS matrix, and **d** magnified SEM image of three-dimensionally interconnected CNT network (*scale bar*: 1 μm)

The surfactant makes it possible to form and maintain air bubbles in the PDMS matrix easily. Figure 2 shows digital and SEM images of the fabricated bubble conductor. Highly roughened surface morphology of the conductor represents the presence of air bubbles entrapped in the conductor, as shown in Fig. 2a. This cross-sectional SEM image in Fig. 2b clearly shows the circular air bubbles inside the conductor. The diameters of the air bubbles were typically in a range from 200 to 500 μm, and the average diameter was ~397.3 μm, as shown in Fig. 2c. We note that the detailed geometry of bubble conductor may be further optimized by controlling the experimental conditions mainly in the stirring and curing steps. Figure 2d shows a magnified SEM image of the CNT networks embedded in the bubble-entrapped PDMS matrix, which suggests that the three-dimensionally interconnected morphologies at the contact junctions provide electrically conductive paths. The electrical resistance of the fabricated bubble conductor was measured to 33.6 ± 7.4 kΩ. Although higher doping level of CNT in the elastic conductor is more suitable for achieving higher electrical conductivity, it may lead to poor processibility due to the increased viscosity of the composites. Therefore, the CNT doping level was experimentally optimized to ~3 wt% with consideration of both the electrical conductivity and the processibility of elastic composites. In addition, it is important to note that the electrical properties of bubble conductor can easily be tailored by doping with various nanomaterials with different electrical conductivity as conductive fillers.

**Fig. 3:**
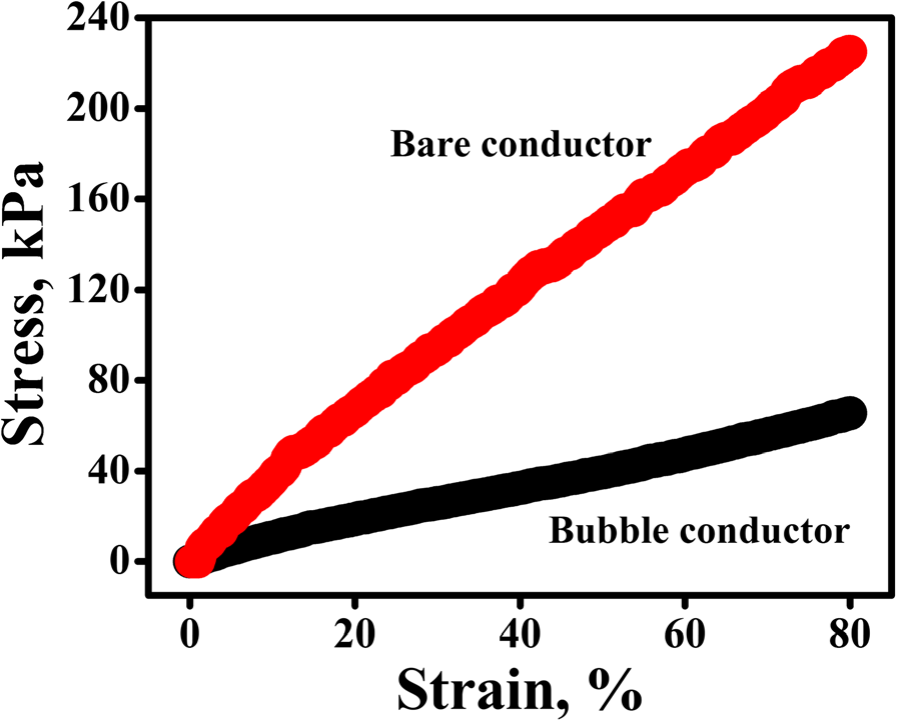
Stress-strain curves of bare conductor and bubble conductor

The effect of air bubbles on the elastic properties of the CNT-doped PDMS films was examined by characterizing the stress-strain relationships of the bare conductor and bubble conductor. Figure 3 shows the stress-strain curves for the stretchable conductors. A stress of ~225 kPa was required to elongate the bare conductor without air bubbles by 1.8 times, which corresponds to ~80 % tensile strain. However, the conductive PDMS film was softened significantly after simply embedding air bubbles into the polymer matrix, as shown in Fig. 3. By making the conductor highly porous, the air bubbles considerably reduce the stress level to ~66 kPa to induce the same strain (80 %). This suggests that the simple bubble formation strategy fairly alleviates increases of the stiffness resulting from toughening in the CNT-reinforced PDMS matrix.

**Fig. 4:**
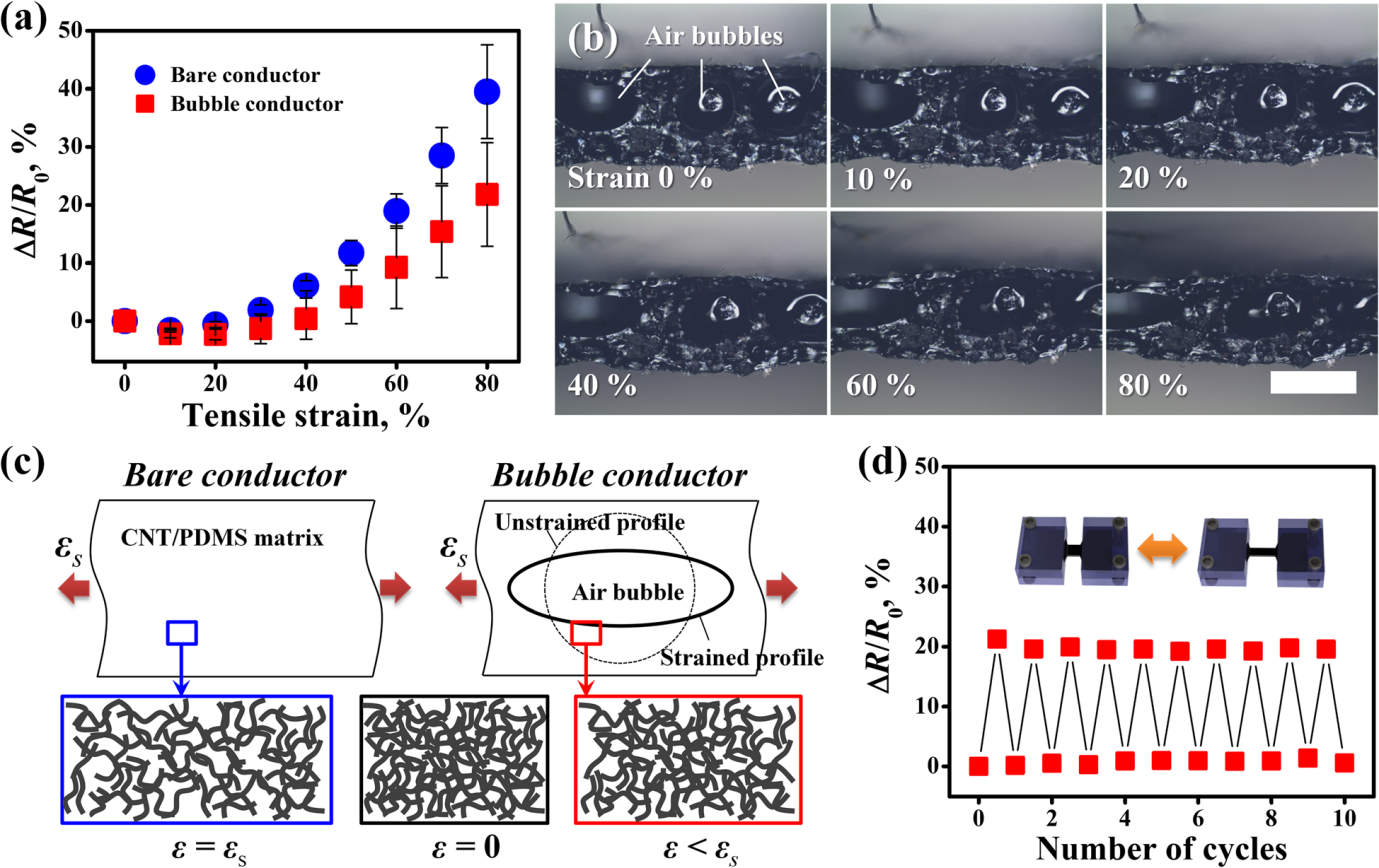
Electrical performance of the fabricated bubble conductor under tensile strain. **a** Δ*R*/*R*_0_ values measured for bare conductor and bubble conductor under tensile strains of up to 80 %, **b** digital cross-sectional images of bubble conductors deformed at different strains (*scale bar*: 500 μm), **c** schematic illustration of effect of air bubbles on the morphologies of the conductive networks in the polymer matrix upon stretching, and **d** Δ*R*/*R*_0_ of bubble conductor under repetitive strain loading (80 %) and unloading (0 %)

Figure 4a shows the changes in the electrical resistance (Δ*R*/*R*_0_) of the fabricated stretchable conductors as a function of the applied tensile strain up to 80 %. Δ*R*/*R*_0_ of the CNT-doped stretchable conductor without air bubbles was only ~6.1 % at a tensile strain of 40 %. This suggests that the three-dimensionally interconnected architecture of the CNT networks in the conductor is greatly helpful for retaining the electrical performance by preventing complete disconnection of the conductive networks, even under relatively high strains.

When gradually increasing the tensile strain, Δ*R*/*R*_0_ of the conductor was increased accordingly due to the resulting decrease in the current paths in the CNT networks, eventually leading to a resistance change of ~39.5 % at a strain of 80 %. The electrical robustness of the stretchable conductor against mechanical deformation was significantly improved by the air bubbles, with a negligible resistance change of ~0.4 % at a strain of 40 %. Moreover, Δ*R*/*R*_0_ of the bubble conductor at a tensile strain of 80 % was decreased by ~44.8 % (from ~39.5 to ~21.8 %) compared to that of the bare conductor. This is mainly attributed to the change in the shape of the air bubbles in response to mechanical deformation.

The air bubbles in the conductor gradually become distorted into an oval shape upon stretching as shown in Fig. 4b. Up to a certain level of strain, most of the CNT networks located at the boundary regions of the air bubbles are just shifted along the deformed profiles of the bubbles rather than experiencing the applied strain directly. Moreover, the inter-CNT contact junctions in the bubble conductor can be maintained better than those of the bare conductor, even under high strains, as illustrated schematically in Fig. 4c. This is probably because that the load applied to the stretchable conductor is inevitably distributed to deform the air bubbles in the polymer matrix. For this reason, Δ*R*/*R*_0_ of the bubble conductor is much smaller than that of the bare conductor at the same strain levels.

In addition, the rate of increase in the electrical resistance of the bubble conductor was also lower than that of the bare conductor, as shown in Fig. 4a. This also means that the morphology of the CNT networks in the bare conductor is greatly affected by the applied tensile strain. In contrast, the air bubbles play an important role in alleviating the resistance change of the device by allowing the CNT networks to retain the networked architecture even at high strains.

Figure 4d shows Δ*R*/*R*_0_ for the bubble conductor under repetitive strain loading (0 to 80 %) and unloading (80 to 0 %) for up to 10 cycles. The Δ*R*/*R*_0_ values were quite uniform for each loading state, representing a minimal standard deviation of ~0.58 %. Importantly, the electrical resistances increased in response to the applied strains and almost returned to the initial state when removing all the strains, as shown in Fig. 4d. In this state, the standard deviation was as small as ~0.36 %. This means that the bubble conductors can operate quite reliably with considerable reversibility even under repetitive high strains.

## Conclusions

We have demonstrated air-bubble-entrapped conductors with both enhanced elastic properties and electrical robustness for stretchable electronics applications. The functional air bubbles can be formed easily and sustained in a CNT-doped PDMS matrix with the aid of a small amount of surfactant. It was experimentally found that the bubble conductor requires ~3.4 times lower stress (~66 kPa) compared to the bare conductor (~225 kPa) to achieve ~80 % elongation. The change in the electrical resistance of the stretchable conductor upon stretching to 80 % was decreased by ~44.8 % (from 39.5 to 21.8 %) by integrating air bubbles into the matrix. The bubble conductor also showed quite reliable and reversible performance under repetitive operations, with low standard deviations of 0.58 and 0.36 % in the loading and unloading states, respectively. The bubble conductors could find many potential applications in stretchable electronics due to the advantages that include simple fabrication, enhanced elastic properties, and electrical robustness against physical deformations.
